# Helical Photonic Metamaterials for Encrypted Chiral Holograms

**DOI:** 10.1002/advs.202507931

**Published:** 2025-07-22

**Authors:** Wonjin Choi, Widianto P. Moestopo, Songyun Gu, Taeil Lee, Jae‐Hyuck Yoo, Xiaoxing Xia, Michael R. Armstrong

**Affiliations:** ^1^ Physical and Life Sciences Materials Science Division Lawrence Livermore National Laboratory Livermore CA 94550 USA; ^2^ Center for Engineered Materials and Manufacturing Lawrence Livermore National Laboratory Livermore CA 94550 USA; ^3^ Materials Science and Engineering Department Gachon University Gyeonggi‐do 13120 Republic of Korea

**Keywords:** chirality‐encoded QR codes, helical structures, terahertz metamaterials

## Abstract

Helical structures are among the most quintessential three‐dimensional (3D) forms that exhibit mirror asymmetry, a hallmark of chirality. Various structural parameters of helices directly linked to chiroptical properties highlight their importance as essential optical metamaterials for polarization‐resolved sensors, imaging, and spectroscopies. However, such function‐defining properties remain incompletely understood due to fabrication challenges and the lack of a relationship between structure and optical properties. Here, helical structures are analyzed parametrically, and correlations are established that are applicable to the design of chiral helical optical metamaterials. By systematically varying independent parameters—such as from single‐turn to five‐turn helices and from small major radii to larger ones optimized to fit the unit cell—the underlying relationships with ellipticty are revealed. In addition to theoretical modeling, the findings are experimentally validated using 3D printing and terahertz spectroscopy. The results demonstrate that optimized helical structures are mechanically tunable and exhibit unprecedented optical properties, including broadband and high‐magnitude ellipticity spectra. Being embedded in soft elastomers, helical arrays can serve as soft, stretchable optical‐mechanical sensors and holograms containing encoded information, such as barcodes and quick response (QR) codes. Chiral QR codes are realized using pixelated single helices with different handedness, demonstrating their potential as advanced encryption/decryption systems for security applications and chiral metaholograms.

## Graphical abstract

This article explores the untapped potential of 3D‐printed helical structures as chiral metamaterials and metaholograms by uncovering how specific geometric parameters govern their chiroptical behavior. Combining terahertz experiments and simulations, it reveals how tunable, stretchable helices enable broadband ellipticity and encrypted QR codes—offering a foundation for next‐generation polarization sensors, soft devices, and secure chiral holography.

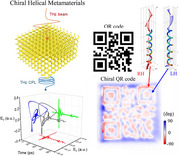

## 1 Introduction

The manipulation of light polarization is one of the fundamental functions of optical elements, achieved through devices such as linear polarizers, gratings, and half‐ and quarter‐wave plates. Polarization optics is critical, as evidenced by the fact that almost all optical devices and imaging systems include at least one polarization element to accurately measure the optical properties of matter.^[^ ^1^, ^2^ ^]^ By utilizing polarization, a single light beam can be split into two based on their polarization state and later recombined after aligning their polarization orientation, enabling the observation of interference patterns. This principle is directly applied in techniques such as differential interference contrast (DIC) microscopy for high‐resolution phase imaging^[^ ^3^, ^4^ ^]^ and quantum photonics for integrating spin states into computations.^[^ ^5^, ^6^ ^]^ Similarly, polarization‐resolved spectroscopies play a crucial role, particularly in circular dichroism (CD) spectroscopy,^[^ ^7^, ^8^, ^9^ ^]^ which is essential for measuring the chirality of nanoparticles, molecules, and molecular assemblies.

Chirality—the property of being non‐superimposable on a mirror image—holds significant importance in biochemistry and pharmaceutical applications, influencing a wide range of processes including enantioselective catalysis and biological interactions such as immune responses.^[^ ^10^, ^11^ ^]^ Moreover, its relevance has expanded to investigations of protein misfolding, providing insights into tertiary and quaternary (bio)structures.^[^ ^8^, ^12^, ^13^, ^14^ ^]^ These measurements are enabled by the broken degeneracy of refractive indices for right‐handed circularly polarized light (RCP) and left‐handed circularly polarized light (LCP) when chiral light interacts with chiral objects.^[^ ^15^ ^]^ The optical activity of molecules has been investigated using electronic circular dichroism (ECD) spectroscopy in the ultraviolet to near‐infrared range and vibrational circular dichroism (VCD) spectroscopy in the near to mid‐infrared range. While the ECD measures the optical activity of electronic transitions primarily from chromophores or metal ions, VCD directly probes the asymmetric vibrations involving a small group of atoms.^[^ ^16^, ^17^ ^]^ More recently, the range of interest has been extended into the far‐infrared region with terahertz (THz) circular dichroism (TCD) spectroscopy, enabling the study of chiral phononic modes in molecules and plasmonic modes in nano‐ to microstructures.^[^ ^8^, ^18^ ^]^

Metamaterials, defined as artificially designed materials consisting of subwavelength meta‐atoms that transcend the limitations of natural materials, are essential for THz optics, as no natural optical crystals, such as quartz or calcite in the visible range, function as wave plates at these frequencies due to the much longer wavelength scales.^[^ ^19^, ^20^ ^]^ CD spectroscopy, anisotropy imaging, and encryption/decryption in THz communication devices.^[^ ^21^, ^22^, ^23^ ^]^ However, most of these approaches are limited by weak optical activity and/or a highly dispersive optical response.^[^ ^24^ ^]^ Specifically, they often fail to achieve full circular phase shifts (λ/4) and exhibit highly resonant, narrowband responses. This limitation is particularly evident in double‐layer‐based chiral structures,^[^ ^25^ ^]^ such as gammadion^[^ ^26^ ^]^ and twisted stacked rods,^[^ ^27^ ^]^ which typically display strong, sharp peaks in transmission and optical activity spectra but with narrow bandwidths. Even continuous 3D structures of chiral origami/kirigami metamaterials,^[^ ^20^, ^22^, ^28^, ^29^ ^]^ despite achieving exceptionally strong optical activity (approaching full circular polarization) and tunability, still face bandwidth limitations (<200 GHz). Conversely, broadband responses can be achieved by incorporating resonance structures of different length scales together, such as a combination of split rings or strips of varying lengths, rather than relying on repetitive units with identical dimensions. However, this approach produces an averaged effective net medium response, balancing the maxima and minima of each resonant mode as expected.^[^ ^30^, ^31^ ^]^ A similar limitation (broad but weak optical activity, ∼10 degrees) is observed when Au strips are deposited via glancing‐angle deposition onto asymmetrically buckled PDMS (polydimethylsiloxane) surfaces.^[^ ^32^ ^]^

Here, we demonstrate that strong, and non‐dispersive, broadband optical activity, including full ellipticity capable of functioning as a quarter‐wave plate in the THz frequency range, can be achieved using 3D‐printed helical arrays. A single helix is a *z*‐stacked array of circularly wound wires that exhibits unique chiral resonances. Additionally, an array of such helices arranged in 2D space demonstrates coupling effects, known as lattice or Bloch resonances, which can be designed and pixelated with various parameters, including opposite handedness.^[^ ^33^, ^34^ ^]^ Nearly all parameters of the helical arrays were tested to determine and elucidate the correlation between their structures and optical properties. Experimental validation was performed using 3D printing, and their capability as chiral bandpass filters or pixelated chiral holograms was tested through the concept of a “chiral” quick response (QR) code, which holds significant potential for chiral display and anti‐counterfeiting as well as encryption and decryption applications.

## 2 Results and Discussion

### 2.1 Optimizing Design and Fabrication of Helical Arrays for THz Applications

**Figure 1:**
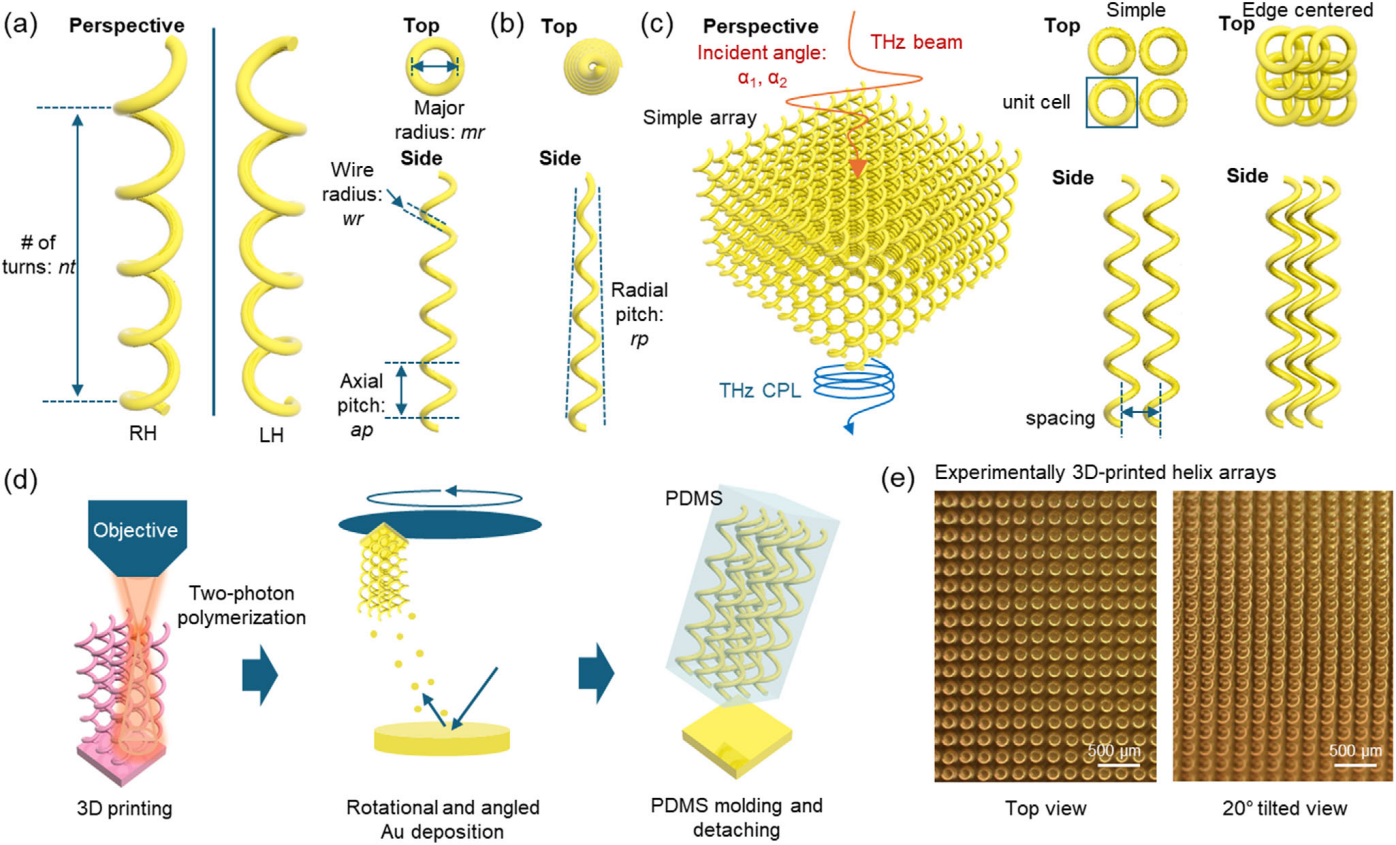
Schematic of helix and helical arrays for THz photonic metamaterials. a) Perspective view of a helix, illustrating the right‐handed (RH) and left‐handed (LH) configurations with mirror symmetry. Key parameters are defined: number of turns (*nt*), wire radius (*wr*), major radius (*mr*), and axial pitch (*ap*); an example is depicted with *nt* = 5, *wr* = 20 µm, *mr* = 75 µm, and *ap* = 250 µm. b) Radial pitch (*rp*) is defined as the radius difference between two consecutive turns of the helix; an example with *rp* = ‐10 µm is shown. c) Helical array configuration and incident angle considerations, including simple and edge‐centered arrangements. d) Schematic of the fabrication process, which involves 3D printing, rotational and angled Au sputtering, and PDMS molding and detachment. e) Optical microscope images of the 3D‐printed structures after Au deposition. Scale bar: 500 µm.

Helical structures are perhaps the most quintessential chiral 3D forms exhibiting mirror asymmetry.^[^ ^35^, ^36^, ^37^ ^]^ While conceptually straightforward, their fabrication involves numerous parameters requiring careful consideration of both mechanical stability and optical function, including major radius (*mr*), wire diameter (*wr*), axial pitch (*ap*), radial pitch (*rp*), number of turns (*nt*), spacing between helices, and the choice between edge‐centered or simple array configurations (**Figure** **1**a–c). While some parameters of helical arrays have been previously studied in the near‐infrared region,^[^ ^38^, ^39^, ^40^, ^41^, ^42^ ^]^ most of these investigations did not directly target the THz frequency range and were therefore not optimized for it. For optical applications of helical arrays, it is particularly important to balance optical activity with mechanical stability and fabrication feasibility. Designs at certain scales may lack the mechanical robustness to stand alone or may pose challenges during fabrication or metal coating.

With advancements in 3D printing, additive methods now enable the fabrication of nearly any helical structure with parameters tailored for the THz frequency range. After printing the polymer structures using two‐photon polymerization (2PP), rotational Au deposition methods were employed, where the entire sample‐loaded plate was rotated. This approach successfully produced Au‐coated helical arrays (Figure 1d,e). It should be noted that Au‐coated polymer helices and fully Au helices (i.e., with no polymer core and composed of entirely of Au) are optically identical, as confirmed by calculations (Figure S1, Supporting Information). Subsequently, the entire printed structure was molded with PDMS (polydimethylsiloxane) and detached from the substrate. This final step is crucial for adapting the arrays into transmissive‐type metamaterials by removing the Au‐coated reflective base. Notably, PDMS exhibits high transparency with a low absorption coefficient, ensuring that only the Au‐coated helical arrays remain active in the THz frequency range (Supporting Figure S2, Supporting Information).^[^ ^43^ ^]^

### 2.2 Simulation‐Based Analysis of Structure‐Chiroptical Relationships

**Figure 2:**
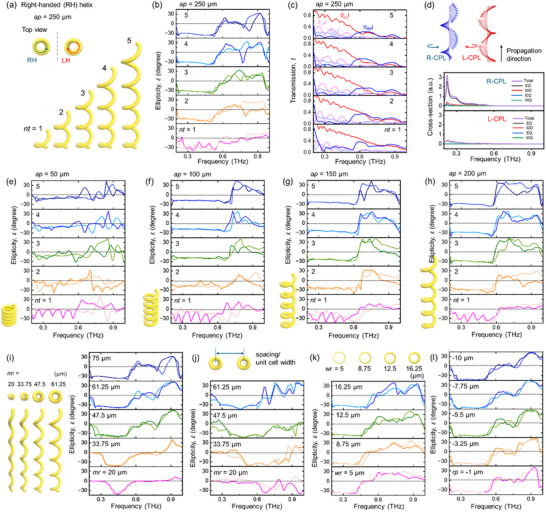
Parametric sweeping of helical array variables. a‐c) Schematic, ellipticity, and transmission results of RH helical (ap = 250 µm) arrays with varying numbers of turns, *nt*. Co‐polarized and cross‐polarized transmission are plotted in (c). d) Multipole decomposition analysis for a helix with *nt* = 5 and *ap* = 250 µm. RCP and LCP light directions are depicted, showing that the electric dipole (ED) is dominant. ED = electric dipole, MD = magnetic dipole, EQ = electric quadrupole, MQ = magnetic quadrupole. e‐h) Ellipticity results of helical arrays with *ap* = 50 to 200 µm and *nt* = 1 to 5. (i, j) Ellipticity of helical arrays with varying mr. i) shows results for a fixed unit cell size, while j) shows results where the unit cell size depends on *mr*. k, l) Ellipticity results for varying *wr* and *rp*, respectively. In each figure, darker spectra correspond to *x*‐polarized input, while brighter spectra correspond to *y*‐polarized input. Slight differences are observed between *x*‐ and *y*‐polarized beams due to the alignment of the starting and ending wire surfaces of the helix with the *x*‐axis.

Various helical arrays with different parameters were investigated using COMSOL, which employs the finite element method (FEM) based on Maxwell's equations (**Figure** **2**). Detailed methods and their validation with previous studies are described in the Materials and Methods section. Throughout the paper, right‐handed (RH) and left‐handed (LH) orientations are defined from the top view of the helix, with the light propagation direction taken into account: anticlockwise for RH and clockwise for LH. All results presented here correspond to RH helical arrays unless otherwise specified.

The first variable for investigating the structure‐optical relationship is the number of turns, *nt*, of the helices, while maintaining an axial pitch, *ap* = 250 µm (Figure 2a–c). Structures with only one turn represent the simplest chiral structure derived from the split‐ring resonator, where one end is elevated by one pitch while the other end is fixed. Even a single turn that breaks the symmetry induces a chiroptical response, with ellipticity showing negative values from 0.2 to 0.5 THz and positive values from 0.5 to 1.2 THz.

Although the response of the ellipticity spectra is not flat, it features multiple chiral peaks, and the transmission of LCP and RCP begins to show a significant difference. As *nt* increases from 1 to 5, the ellipticity band not only becomes flatter but also slightly increases in magnitude. This trend in *nt* is consistent with previous work.^[^ ^33^, ^38^ ^]^ Further investigation revealed that symmetry breaking begins to emerge at approximately *nt* = 1/2, and becomes quite noticeable from *nt* = 2/3 (Figure S3, Supporting Information). To better understand this behavior, we analyzed the decomposed transmission spectra using co‐polarized and cross‐polarized components (Figure 2c). Similar to earlier reports,^[^ ^44^, ^45^ ^]^ our results show that over a broad range of resonance frequency range (0.2–0.6 THz), the co‐polarized components |*t_LL_* | and |*t_RR_* | exhibit opposite optical responses, indicating a strong chiral activity. Additionally, we observe slight circular polarization conversion (|*t_LR_* | and |*t_RL_* |).^[^ ^46^ ^]^ This behavior was further investigated through multipole decomposition analysis (Figure 2d),^[^ ^47^, ^48^ ^]^ which revealed that the lower |*t_RR_* | compared to |*t_LL_* | is primarily caused by strong electric induction—along with contributions from magnetic dipoles and quadrupoles—under RCP illumination at frequencies below 0.6 THz (e.g., 0.3 THz), and under LCP illumination ≈0.71 THz. These trends are further supported by the near‐field electric field distribution maps (Figure S4, Supporting Information), which show minimal Poynting vector flow or power flow at 0.3 and 0.71 THz for RCP and LCP, respectively.

Simulations with *ap* less than 250 µm were also performed for different *nt* to investigate the relationship between *ap* and ellipticity, as well as to identify optimized conditions for the designed frequency of the application (Figure 2e–h). The results clearly show that a smaller axial pitch (*ap*) along the light propagation direction covers a broader frequency range. For instance, when *ap* = 50 µm, the structure spans over 0.5 THz. However, the magnitude of ellipticity is relatively lower (≈12 degrees) compared to helices with larger *ap*. Since the wire radius (*wr*) is set to 20 µm, *ap* values below 40 µm are not feasible due to structural overlap. As observed in the case of *ap* = 250 µm, increasing *nt* similarly results in a flatter ellipticity profile and a slight increase in magnitude.

The *mr* of the helix is another critical parameter, as it determines the degree of twisting, i.e., the curvature of the wires per cycle, and significantly impacts the resonant frequency range (Figure 2i). In Figure 2a–h, all helices use *mr* = 75 µm; here, *mr* was parametrically varied from 20 µm in steps of 13.75 µm while maintaining *nt* = 5, *ap* = 250 µm, and *wr* = 20 µm.

When the helix has the smallest radius (*mr* = 20 µm), the frequency range showing negative ellipticity is limited to 0.4–0.5 THz. As *mr* increases, this range broadens, indicating that higher‐frequency THz beams correspond to chiral plasmonic resonances in structures with smaller *mr*, while lower frequencies (i.e., longer wavelengths) interact more effectively as *mr* becomes larger. However, caution is needed in interpreting the effect of *mr*, as the spacing between helices in this case was fixed during the variation of *mr* from 75 to 20 µm to maintain the unit cell box size. For the above cases, the spacing between helices was set to 200 µm, considering *wr* = 20 µm and *mr* = 75 µm, which results in a precise PDMS gap of 10 µm between adjacent helices. To explore the effect of varying *mr* while maintaining a constant 10 µm gap between helices (i.e., when the unit cell size is linked to *mr*), simulations were conducted, and the results are shown in Figure 2j. These simulations reveal that while the overall trend remains consistent, the active frequency exhibits a blueshift due to coupling between adjacent helices; this shift is more pronounced in configurations with smaller unit cell sizes.

Other parameters were also considered to assess their effects on the ellipticity of the helical arrays. Although it is challenging to 3D print helical arrays with thinner wire diameters due to structural instability during the development and rinsing processes (as observed in our experimental attempts), simulations were conducted and revealed relatively minor differences in ellipticity (Figure 2k). Additionally, the *rp* of the helix was investigated. This parameter is defined as the difference between the major radii of each successive turn of the helix. For example, if *rp* = −10 µm, and the *mr* of the first cycle at the bottom is 75 µm, the *mr* of the fifth cycle at the top becomes 35 µm, as illustrated in Figure 1b. The typical trend observed in this case also holds true for the inverted version of the helix with *rp*, where the beam is introduced in the opposite direction (Figure S5, Supporting Information). While variations in *rp* produced relatively minor effects on the ellipticity, the results revealed an important insight: overall spectral shaping and the resonant frequency range are determined by the helical structures as a whole, irrespective of the direction from which the light is incident. This conclusion is further supported by the normalized electric field distribution maps shown in Figure 4b. Additionally, various other methods—such as textile manufacturing techniques and wire helix formation on wavy or corrugated surfaces with metal ribbon patterns—have also been proposed for the sub‐THz or THz frequency range.^[^ ^49^, ^50^ ^]^ However, most of these methods, aside from 3D printing, cannot produce vertically aligned helical arrays, which limit their ability to achieve broad ellipticity over a wide frequency range. In particular, the alignment of the beam propagation direction along the height (*z*‐axis) of the helical arrays is a crucial factor for achieving strong and broadband ellipticity—that is, it is dependent on the incident angle.

Based on computational results for effective THz modulation with helical arrays, we fabricated helical arrays embedded in PDMS that exhibited both broad and high magnitude ellipticity. Helices with parameters of *ap* = 250 µm, *nt* = 5, *mr* = 75 µm, *wr* = 20 µm, and *rp* = 0 µm were selected for optimized optical performance, mechanical stability, and compatibility with all the chemical processes and PDMS molding/lift‐off procedures.

### 2.3 Experimental Validation and Chiroptical Responses of Helical Metamaterials

**Figure 3:**
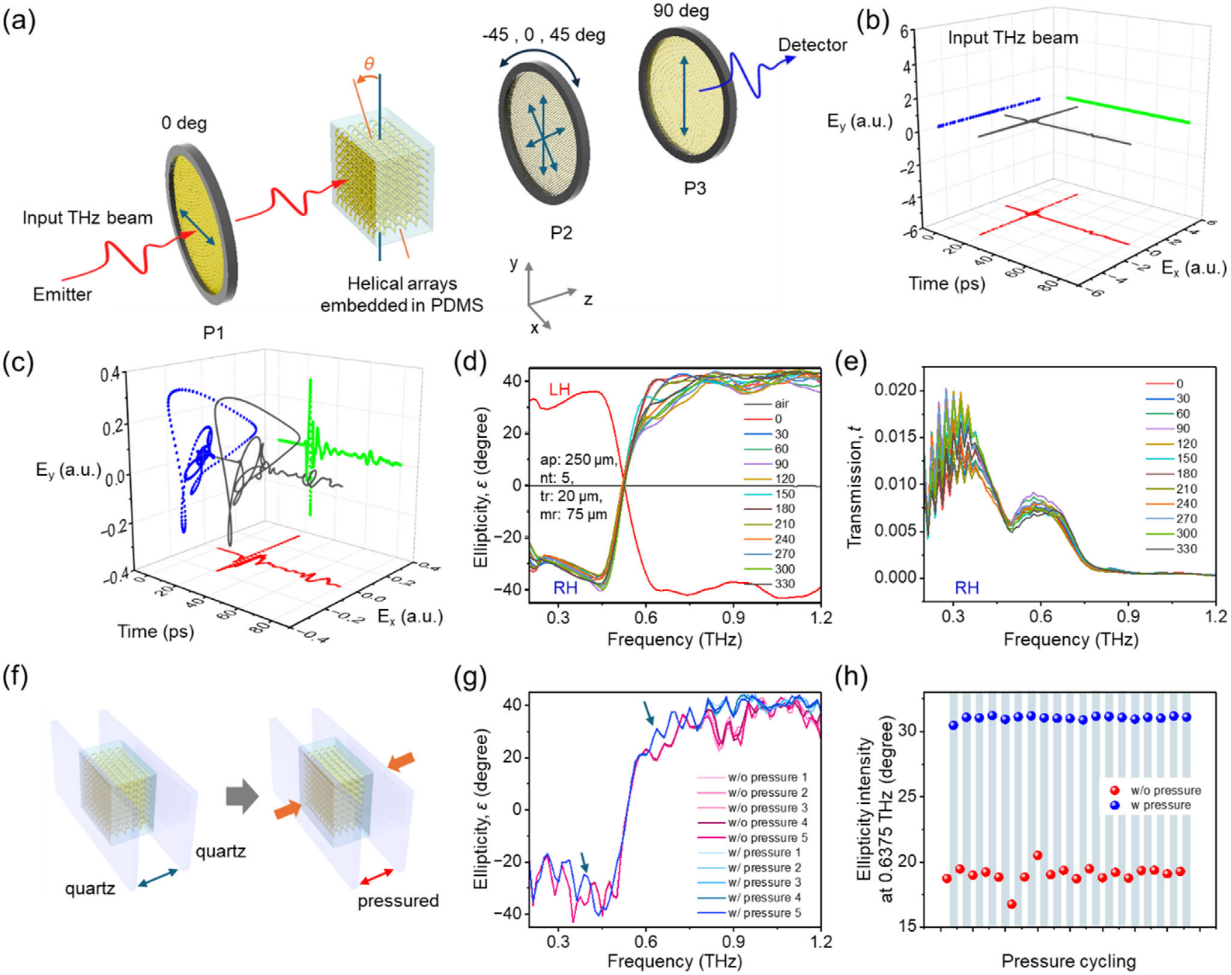
THz time‐domain polarimetry measurements of helix array metamaterials. a) Schematic of the THz‐TDS measurement setup with three linear polarizers (P1‐P3, representing THz wire grid polarizers). The samples were measured with respect to the azimuthal rotational angle, *θ*, to investigate angle dependence and structural uniformity. b) Results for the reference input beam, which is *x* polarized. The *E_y_* component shows near‐zero magnitude throughout the time range of interest. c) After passing through the helix array metamaterials, the *E_y_* component is observed to have a magnitude similar to that of *E_x_*, indicating the clear optical rotatory power of the metamaterials, as evidenced by the intricate temporal evolution of the *E_y_* component. d) Ellipticity spectra of LH and RH helical arrays. For the RH sample, azimuthal angle dependence was measured. e) Transmission of RH helical arrays at different *θ*, showing high consistency. f) The effect of mechanical pressure was investigated, with a schematic illustrating the sample before and after pressure application. g) Ellipticity spectra for five representative samples, comparing pristine and pressurized states. h) Cycling of pressure and release of the sample was recorded, showing high fidelity, particularly at 0.6475 THz, consistent with simulation results.

For measurements, THz time‐domain spectroscopy with three linear polarizers (P1, P2, and P3) was employed. P1 and P3 were fixed in a cross‐polarized configuration, while P2 was automatically rotated to three states: 45°, 0°, and 45° (**Figure** **3**a). Detailed measurement methods are described in the Supporting Information. Prior to measuring the optical properties of the helical metamaterials, reference air data confirmed that the THz beam was linearly polarized along the *x*‐axis (pure *E_x_* field) through three measurements and subsequent calculations (Figure 3b). After introducing the sample at the focal point, the *x*‐polarized input beam transformed into a complex signal in 3D space within the time‐domain spectrum, exhibiting *E_x_* and *E_y_* fields of nearly comparable magnitudes (Figure 3c). It should also be noted that when ellipticities are measured using both *x*‐polarized and *y*‐polarized input beams, THz CD or TCD can be calculated from these measurements using the Jones matrix elements (Figure S6, Supporting Information).

Fast Fourier Transform (FFT) analysis allowed the calculation of ellipticity using Stokes parameters derived from the *Ex* and *Ey* fields. The results revealed an almost full ellipticity of ≈40° from 0.2 to 0.5 THz, transitioning from negative to positive and remaining positive beyond 0.6 THz. Remarkably, experimental results matched the *ab ovo* FEM calculations almost perfectly for both ellipticity and transmission (Figure 3d,e). In the transmission data shown in Figure 3e, considering that the noise floor is on the order of 10^−10^, this level of transmission is not considered low for our THz‐TDS system. Additionally, by calculating the transmittance under RCP and LCP illumination, we observe a handedness‐dependent behavior that is in strong agreement with the measured ellipticity (Figure S7, Supporting Information). Furthermore, as expected, the azimuthal rotational angle (*θ*) of the helical arrays had a negligible effect on the spectra, confirming the almost perfect rotational symmetry and geometric intrinsic chirality (Figure 3d). It should be noted that, in many cases, structures with extrinsic chirality or linear bianisotropy can produce slightly different spectra when rotated and, in some cases, even reverse the sign.^[^ ^51^ ^]^ We also observed slight discrepancies between simulation and experimental results, with experimental ellipticity values being higher in some regions. These differences may arise from variations in *ap* during fabrication or slight inaccuracies in the *n* and *k* values used for PDMS in the simulations. The impact of these parameters is further illustrated in the Figure S8 (Supporting Information).

Helical arrays serve as optical metamaterials and can also be viewed as microscale mechanical springs, where the spacing between helices can be tuned by applying stretching or compressing forces in the *x* or *y* direction, and the *ap* can be altered by applying pressure along the *z*‐axis (Figure 3f). To apply a uniform force across the surface, two THz‐transparent quartz slide glasses were placed on the front and back of the PDMS‐embedded helical arrays, enabling the investigation of pressing and releasing pressure cycles (Figure 3g,h). Approximately 6.67% strain was applied along the sample thickness direction, i.e., resulting in a reduction in height or axial pitch of the helices. Consistent with predicted simulation trends, the application of pressure caused a slight increase in the magnitude of the negative ellipticity (<0.5 THz) and a significant increase in the positive ellipticity, particularly around 0.7 THz. This behavior closely mirrored the trend observed when *ap* was reduced from 250 to 200 µm. Note that this trend differs from blue‐shift behavior observed when the axial pitch of cholesteric liquid crystals changes under compression, as the total length of the helix here is not physically altered. The effect was most pronounced when tracking the ellipticity value at 0.7 THz during pressure/release cycling. The ellipticity demonstrated a highly reproducible response with high cyclic fidelity (Figure 3h).

### 2.4 Chiral QR Codes for Polarization‐Based Encryption

A compelling question that could arise here is whether a single helix can induce optical activity comparable to that of an array of helices. To address this, we compared the ellipticity spectra of a single helix with those of helical arrays and found that, although similar optical rotation and ellipticity features were observed, their magnitudes were significantly smaller in the single helix, implying that the broadband ellipticity magnitude is enhanced by inter‐helix coupling (Figure S9, Supporting Information). This result highlights the intriguing potential of these helical metamaterials, even as single helices, for pixelated applications like chiral holograms, spatially varied band‐pass filters, or wavefront engineering films for anti‐counterfeit purposes.

**Figure 4:**
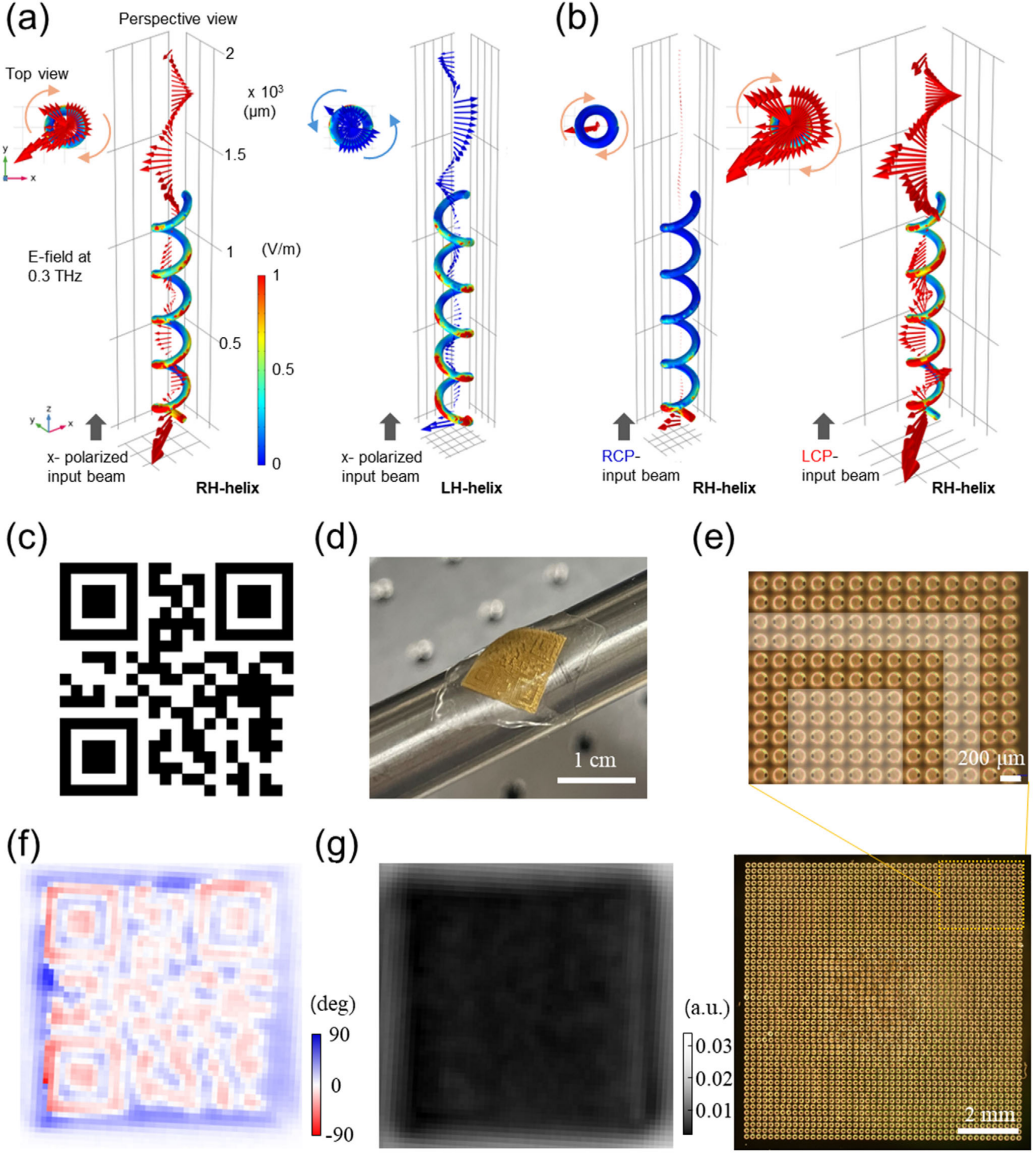
Single‐helix single‐pixel metaholograms. a) Propagation of a linearly polarized THz beam through single helices of LH and RH. The induced electric field distribution is depicted on the surface of the helices using color, while the field in the surrounding space is represented by arrows. b) Band‐pass filter behavior of helices for circularly polarized input light. c) Designed QR code representing 'C‐QR’. d) Photograph of a flexible chiral QR code attached to a 0.5" optical post. e) Optical microscope images of the chiral QR helices. f) Polarization rotation angle map at 1 THz for the chiral QR, measured using THz‐TDS. g) Transmission map at 1 THz for the chiral QR.

To explore the feasibility and concept, we first examined the THz beam propagation behavior through a single helix (Figure. 4a,b; Movies S1 and S2, Supporting Information). When a linearly polarized beam (*x*‐pol.) enters from the bottom of the unit cell, it is converted into RCP (for RH helices) or LCP (for LH helices), functioning similarly to a circular polarizer or quarter waveplate (**Figure** **4**a).^[^ ^52^ ^]^ The helix acts not only as a circular polarizer but also as a circular band‐pass filter, where the RH helix transmits only the LCP beam while blocking almost all co‐directional beams (Figure 4b). These results also align well with previous findings obtained in the microwave range.^[^ ^53^ ^]^ Notably, most beams with the same handedness are extinguished by the first (bottom) turn of the helix, as indicated by the high magnitude of the induced electric field.

Among the potential applications of chirally pixelated holograms, we selected QR codes to incorporate chirality, creating unique ‘chiral QR’ codes with applications in anti‐counterfeiting and protecting personal information. By adding an additional data dimension to barcode, QR codes remain one of the most convenient methods for encoding information, making them ubiquitous today. However, a significant issue limiting their direct adoption for sensitive applications is their lack of security. For instance, personal and banking information encoded in visible QR codes can be easily accessed without permission, whereas chirality‐encoded QR codes can serve as keys for secure deciphering.

Here, we showcase a chiral QR code made of helices, where only the correct frequency and handedness of light reveal the encoded information (Figure 4c–g). Figure 4c shows the design of the chiral QR pattern, along with a photo of the sample after fabrication and the lift‐off process (Figure 4d). Embedded in PDMS, this flexible chiral QR code can be attached to surfaces with various curvatures. Optical microscope images (Figure 4e) of the entire structure, along with zoomed‐in views, show near‐perfect fabrication of pixels with two distinct handedness helices: LH helices for black pixels and RH helices for white pixels, respectively. THz polarimetry results indicate that the map of polarization rotation angle matches the intended design, while the THz absorption reveals an indistinguishable image, highlighting the need for a proper chiral decryption system to decode the QR code (Figure 4f,g). It should be noted that while we use visible‐range transparent PDMS as a host, using opaque rubber would render it invisible but still functional in the THz range.

## 3 Conclusion

In conclusion, the chiroptical properties of helical arrays, one of the most inherently chiral structures, were investigated both theoretically and experimentally, primarily in the THz frequency range. Notably, a broad and flat response in ellipticity and TCD was observed, particularly for helices with multiple turns. This broadband and high‐magnitude ellipticity arises from their intrinsic chirality, multiple resonance pathways (intra‐ and inter‐helix coupling), asymmetric interaction with circularly polarized light, and structural dispersion—all of which combine to enable strong and wideband chiral light–matter interactions. This characteristic opens up opportunities for diverse applications, including metaholograms, band‐pass filters for 5G/6G telecommunications, and advanced encryption and decryption systems. Additionally, single helix–single pixel holograms were explored, showcasing their unique potential for chiral QR code applications. These codes, which leverage the lock‐and‐key principle, can securely encode information, providing an innovative approach to anti‐counterfeiting and secure data storage. By combining the versatility of chiral structures with their frequency‐selective optical properties, this work highlights a path forward for functional chiral metamaterials across a wide range of fields.

## Conflict of Interest

The authors declare no conflict of interest.

## Supporting information



Supporting Information

Supplemental Movie 1

Supplemental Movie 2

## Data Availability

The data that support the findings of this study are available from the corresponding author upon reasonable request.
