# Supplementary material for: Helical Photonic Metamaterials for Encrypted Chiral Holograms
Source: Adv Sci (Weinh). 2025 Jul 22;12(38):e07931. doi: 10.1002/advs.202507931 (PMC12520478; doi:10.1002/advs.202507931)
Supplement: Supplementary file 1 — Supporting Information [file ADVS-12-e07931-s001.docx]

Supporting Information

Helical Photonic Metamaterials for Encrypted Chiral Holograms

*Wonjin Choi*, Widianto Moestopo, Songyun Gu, Taeil Lee, Jae-Hyuck Yoo, Xioxing Xia, and Michael R. Armstrong*

**This PDF file includes:**

Materials and Methods

Figs. S1 to S9

References

**Other Supplementary Materials for this manuscript includes:**

Supplementary Movies S1, S2

**Methods**

Fabrication of 3D-printed helix arrays

We utilized a commercial two-photon polymerization (2PP) 3D printer system (NanoOne 1000, UpNano GmbH) equipped with a 10x objective (UPLXAPO10X, Olympus) and a 780 nm-wavelength femtosecond laser to manufacture “Simple” 75 x 75 arrays of micron-sized helixes out of a commercial resin (UpPhoto, UpNano GmbH). The helix arrays and the base underneath them were printed together using standard coarse printing parameters (100 mW laser power, 600 mm/s scan speed, 5 µm layer height, and 4.2 µm coarse line distance). Once printed and rinsed in PGMEA and IPA baths, the helix arrays were coated with 1 µm-thick Au layer using an M3 E-Beam system with a 60-degree sample tilt, a Ti adhesion layer set at 150 A, and a KLA-Tencor D-600 profilometer for Au layer thickness measurement. The helix arrays were then infilled with PDMS followed by detachment from the printed base for characterization once the PDMS cured.

Fabrication of chiral QR code using different handedness helices

The helix QR code was produced using a non-disclosed custom 2PP system and photo-resin with >2000 femtosecond laser focal spots printing in parallel. The LH and RH helices are printed by actively turning on and off the selected focal spots during scanning the toolpath (1.5 µm layer height, 0.2 µm line distance). After printing, the structure was developed in PGMEA and IPA to remove excessive resin, then critical-point dried (Autosamdri®-931, tousimis research corporation) to prevent structure distortion from liquid drying. The following Au coating and PDMS embedding processes are the same as described above for helix arrays.

Finite-element modeling for electromagnetic simulations

All simulations, including ellipticity, transmission, current norm distribution, and multipole decomposition analysis, were conducted using finite element simulation software (COMSOL Multiphysics 6.3, COMSOL Inc.) with the helix model. Periodic boundary (Floquet) conditions were applied to a single unit cell of each helix model. A boundary box of 200 µm × 200 µm was typically used, except in cases where the unit cell size was varied relative to the major radius size (Fig. 2j). Transition boundary conditions were employed for the metallic layer of the helix to reduce computational costs. The electrical conductivity and thickness were set to 45.6×10^6^ S/m and 1 µm, respectively, in accordance with experimental data. The real and imaginary parts of the refractive index of PDMS were 1.6 and 0.04, respectively, as experimentally determined in Ref. ^[1]^

The side surfaces of the unit cell box were assigned periodic boundary conditions, while the bottom and top surfaces served as port surfaces, with the bottom functioning as the input and the top as the output. For ellipticity and transmission calculations, the surface-averaged electric field components ($E_{x}$​ and $E_{y}$​) were obtained from the top surface. The same equations used in experimental data analysis were applied to ensure consistency.

Electric field norm distributions and arrow plots were generated using the 2D plot group under the "Electric Field (*emw*)" function. Multipole decomposition analysis was performed following the equations outlined in reference^[2,3]^.

THz-TDS measurements

Terahertz time-domain spectroscopy (THz-TDS) with three linear polarizers was used to measure the optical properties of the helical metamaterials (Menlo Systems, TETA K15). The Tera15-FC antenna module (Menlo Systems), which uses photoconductive antennas and a pulsed laser (<90 fs pulse duration, 100 MHz repetition rate) at 1560 nm, served as both the THz emitter and detector. THz lenses (TPX50, Thorlabs) made of polymethylpentene, with a diameter of 1.5 inches and a focal length of 50 mm, were used to produce collimated and focused THz beams via two 4f systems. Raster scanning was achieved using a motorized two-axis stage system (M-403 Linear Stages, PI Instrument). Samples were mounted on an automatically controllable rotational mount to measure the complete 360-degree azimuthal angle orientation dependence. The entire system was enclosed within custom-designed acrylic boxes to facilitate nitrogen purging.

Three THz wire grid polarizers (G30 × 10-S, Microtech Instruments, Inc.) with an extinction ratio of 10^4^ in the spectral range of 0.2 – 2.5 THz were utilized, as described elsewhere^[4]^. The chiroptical responses of the helical metamaterials were measured using a setup in which the orientation of the linearly polarized THz emitter beam was fixed horizontally (*x*-axis) relative to the optical breadboards, while the THz detector was fixed vertically (*y*-axis).

The setup consisted of three polarizers (P1, P2, and P3, as described in Figure 3). P1 was placed in front of the emitter, with its transmission direction, perpendicular to the wire grid orientation, fixed to the *x*-axis to ensure that the input beam was linearly polarized. P3 was positioned just before the THz detector, with its transmission direction fixed to the *y*-axis, making it cross-polarized relative to P1 as well as the emitter. P2 was placed between P1 and P3 and was rotated to various orientations to determine the complete polarization states of the transmitted electric field^[4,5]^.

For the measurement of QR images, the image (Figure 4(f)) was obtained using THz imaging with a motorized x–y scanning stage. The sample was positioned at the focal point of the THz lenses, and the x–y stage was integrated with the THz-TDS system to measure three different polarization states at each pixel. The step size in both the x and y directions was 200 μm, resulting in a total of 66 × 66 pixels, each containing the full THz spectrum.

The calculation of chiroptical properties begins with calculating the *x-* and *y-*components of the electric field. The *x*-component of the electric field, $E_{x}\left( t \right)$, is calculated using Equation (1), where $E_{+45^{\circ}}\left( t \right)$ and $E_{-45^{\circ}}\left( t \right)$ are the time-domain electric fields measured for the two polarizer orientations of the P2, set at +45° and -45° relative to the *y*-axis, respectively. Conversely, $E_{y}\left( t \right)$ can be directly obtained when P2 is set to 0°. Figure 3b and 3c show direct results of these two fields in the time domain.

$$\begin{aligned} E_{x}\left( t \right)=E_{+45^{\circ}}\left( t \right)-E_{-45^{\circ}}\left( t \right)\#\left( 1 \right) \end{aligned}$$

The fast Fourier transform (FFT) is then used to obtain complex frequency-domain electric field spectra from the time-domain electric fields measured, as shown in Equations (2).

$\tilde{E}_{x}=\tilde{E}_{x}\left( \omega\right)=FFT\{E_{x}\left( t \right)\}$

$$\begin{aligned} \tilde{E}_{y}=\tilde{E}_{y}\left( \omega\right)=FFT\{E_{y}\left( t \right)\}\#\left( 2 \right) \end{aligned}$$

Once $\tilde{E}_{x}$ and $\tilde{E}_{y}$ are obtained, the Stokes equations are used to calculate the ellipticity and polarization rotation angle, as follows:

$$S_{0}=\tilde{E}_{x}\tilde{E}_{x}^{*}+\tilde{E}_{y}\tilde{E}_{y}^{*}$$

$$S_{1}=\tilde{E}_{x}\tilde{E}_{x}^{*}-\tilde{E}_{y}\tilde{E}_{y}^{*}$$

$$S_{2}=\tilde{E}_{x}\tilde{E}_{y}^{*}+\tilde{E}_{y}\tilde{E}_{x}^{*}$$

$$\begin{aligned} S_{3}={i(\tilde{E}}_{x}\tilde{E}_{y}^{*}-\tilde{E}_{y}\tilde{E}_{x}^{*})\#\left( 3 \right) \end{aligned}$$

The polarization rotation angle (*θ*) and ellipticity (*η*) are defined by Equations (4), where $-\frac{\pi}{2}\leq\theta\leq\frac{\pi}{2}$ and $-\frac{\pi}{4}\leq\eta\leq\frac{\pi}{4}$.

$$\theta=\frac{1}{2}tan^{-1}\left( \frac{S_{2}}{S_{1}} \right)$$

$$\begin{aligned} \eta=\frac{1}{2}sin^{-1}\left( \frac{S_{3}}{S_{0}} \right) \#\left( 4 \right) \end{aligned}$$

Transmission and absorption are calculated here using the magnitude of the total electric field, combining both the *x*- and *y*-components.

$T=\sqrt{\left| \tilde{E}_{x} \right|^{2}+\left| \tilde{E}_{y} \right|^{2}}$ (5)

$\alpha=-\frac{1}{d}ln(\frac{T_{sample}}{T_{ref}})$ (6)

where, *d* is the thickness of the sample.

The transmitted electric field for a circularly polarized incident beam can be inferred using the Jones matrix elements measured from linearly polarized incident fields. For a normalized right circularly polarized (RCP) incident beam

$$\begin{aligned} \tilde{E}_{RCP}^{in}=\frac{1}{\sqrt{2}}\binom{1}{i}\#\left( 7 \right) \end{aligned}$$

the electric field of the transmitted wave is

$$\begin{aligned} \tilde{E}_{RCP}^{out}=\left( {t_{xx} \atop t_{xy}}{t_{yx} \atop t_{yy}} \right)\frac{1}{\sqrt{2}}\binom{1}{i}=\frac{1}{\sqrt{2}}\binom{t_{xx}+it_{yx}}{t_{xy}+it_{yy}}\#\left( 8 \right) \end{aligned}$$

and the magnitude of this complex electric field vector is

$$\begin{aligned} E_{RCP}=\frac{1}{\sqrt{2}}\sqrt{\left| t_{xx}+it_{yx} \right|^{2}+\left| t_{xy}+it_{yy} \right|^{2}}\#\left( 9 \right) \end{aligned}$$

where $\left| \right|$ is the absolute value of a complex number.

Similarly, for a normalized left circularly polarized (LCP) incident beam

$$\begin{aligned} \tilde{E}_{LCP}^{in}=\frac{1}{\sqrt{2}}\binom{1}{-i}\#\left( 10 \right) \end{aligned}$$

the electric field of the corresponding transmitted wave is

$$\begin{aligned} \tilde{E}_{LCP}^{out}=\left( {t_{xx} \atop t_{xy}}{t_{yx} \atop t_{yy}} \right)\frac{1}{\sqrt{2}}\binom{1}{-i}=\frac{1}{\sqrt{2}}\binom{t_{xx}-it_{yx}}{t_{xy}-it_{yy}}\#\left( 11 \right) \end{aligned}$$

and the magnitude of this complex electric field vector is

$$\begin{aligned} E_{LCP}=\frac{1}{\sqrt{2}}\sqrt{\left| t_{xx}-it_{yx} \right|^{2}+\left| t_{xy}-it_{yy} \right|^{2}}\#\left( 12 \right) \end{aligned}$$

The THz CD or TCD is the relative transmission (or absorption) difference between RCP and LCP incident waves, and can be defined and quantified by

$$\begin{aligned} TCD =tan^{-1}\left( \frac{E_{LCP}-E_{RCP}}{E_{LCP}+E_{RCP}} \right)\#\left( 13 \right) \end{aligned}$$

where $E_{RCP}$ and $E_{LCP}$ are the magnitudes of the transmitted waves of RCP and LCP incident beams given by Eq. (4) and Eq. (7), respectively.

Decomposed transmission spectra for co- and cross-polarzied is calculated based on following equations.

$t_{LL}=\frac{1}{2}(t_{xx}+t_{yy}+i\left( t_{xy}-t_{yx} \right))$ (14)

$t_{LR}=\frac{1}{2}\left( t_{xx}-t_{yy}+i\left( t_{xy}+t_{yx} \right) \right)$ (15)

$t_{RL}=\frac{1}{2}\left( t_{xx}-t_{yy}-i\left( t_{xy}+t_{yx} \right) \right)$ (16)

$t_{RR}=\frac{1}{2}(t_{xx}+t_{yy}-i\left( t_{xy}-t_{yx} \right))$ (17)


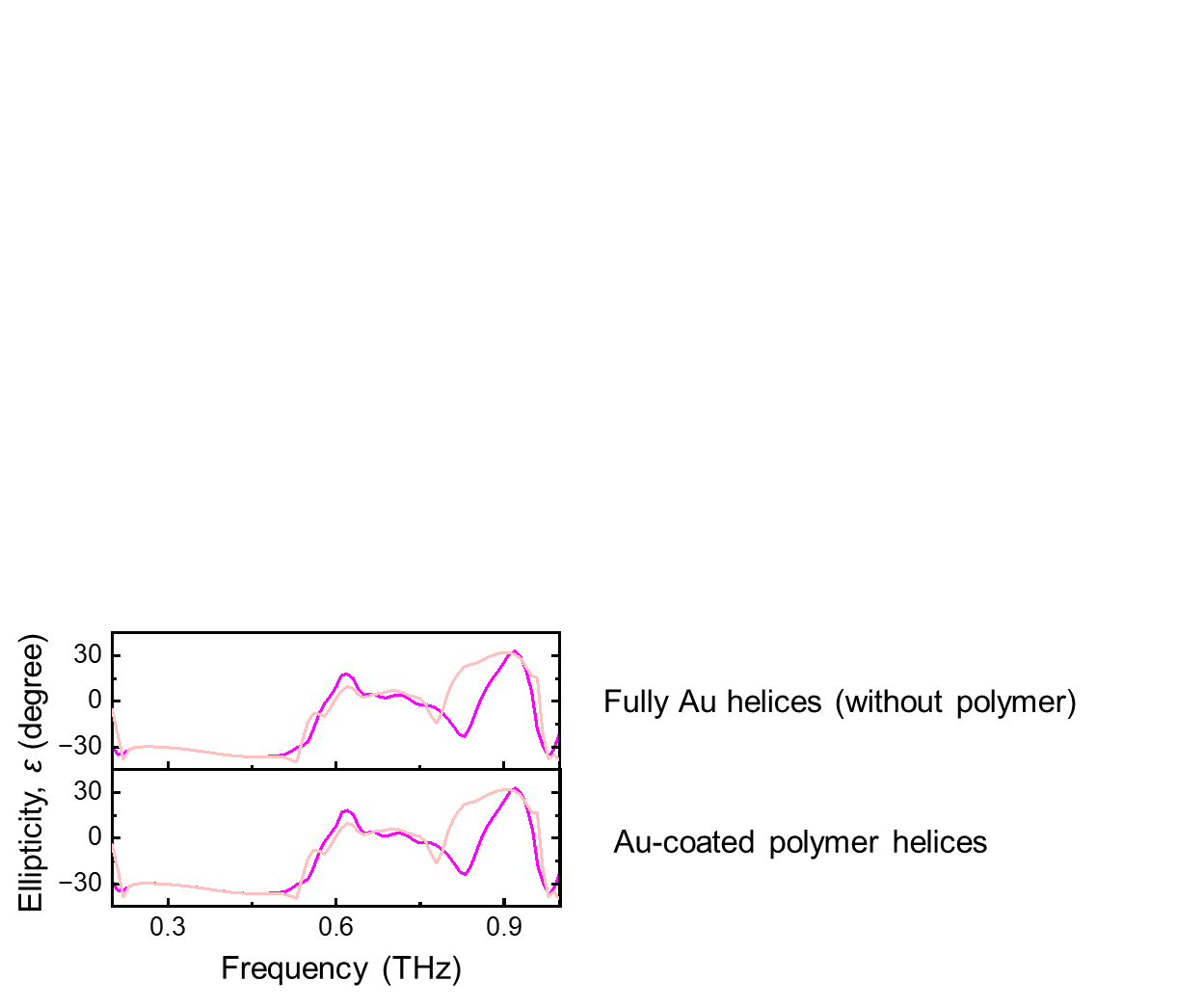


**Figure S1. Ellipticity results for fully Au helices and Au-coated polymer helices.** For the computations, data for fully Au helices were derived from the Au domain, while the Au-coated helices feature a 1 µm thick Au shell (transition layer) with a polymer core. As THz light cannot penetrate the 1 µm thick Au layer, fully Au helices and Au-coated helices exhibit identical optical responses, behaving as optical twins.

**Figure S2.** Measured absorption coefficient of PDMS, showing a low absorption (< 5 cm⁻¹) and a dispersionless characteristic across the entire frequency range.


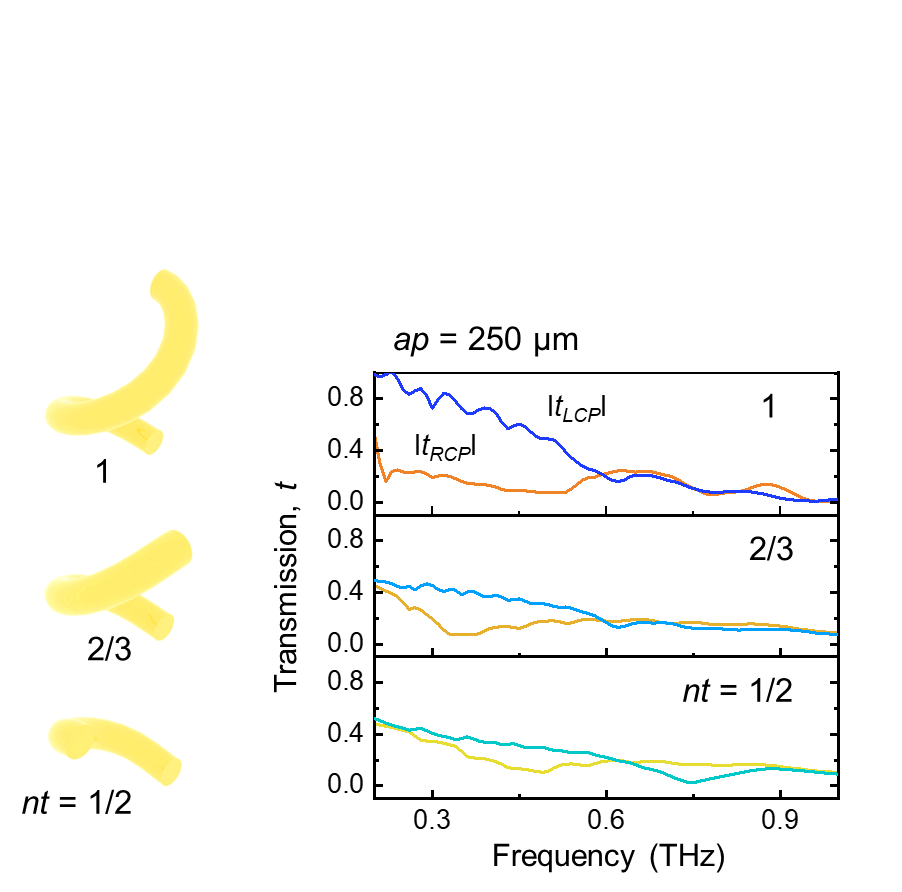


**Figure S3.** Simulated transmission spectra of RH with *ap* = 250 μm and *nt* < 1, illustrating the onset of symmetry breaking. The diverge between LCP and RCP transmission begins to emerge around *nt* = 1/2 and becomes more pronounced from *nt* = 2/3.


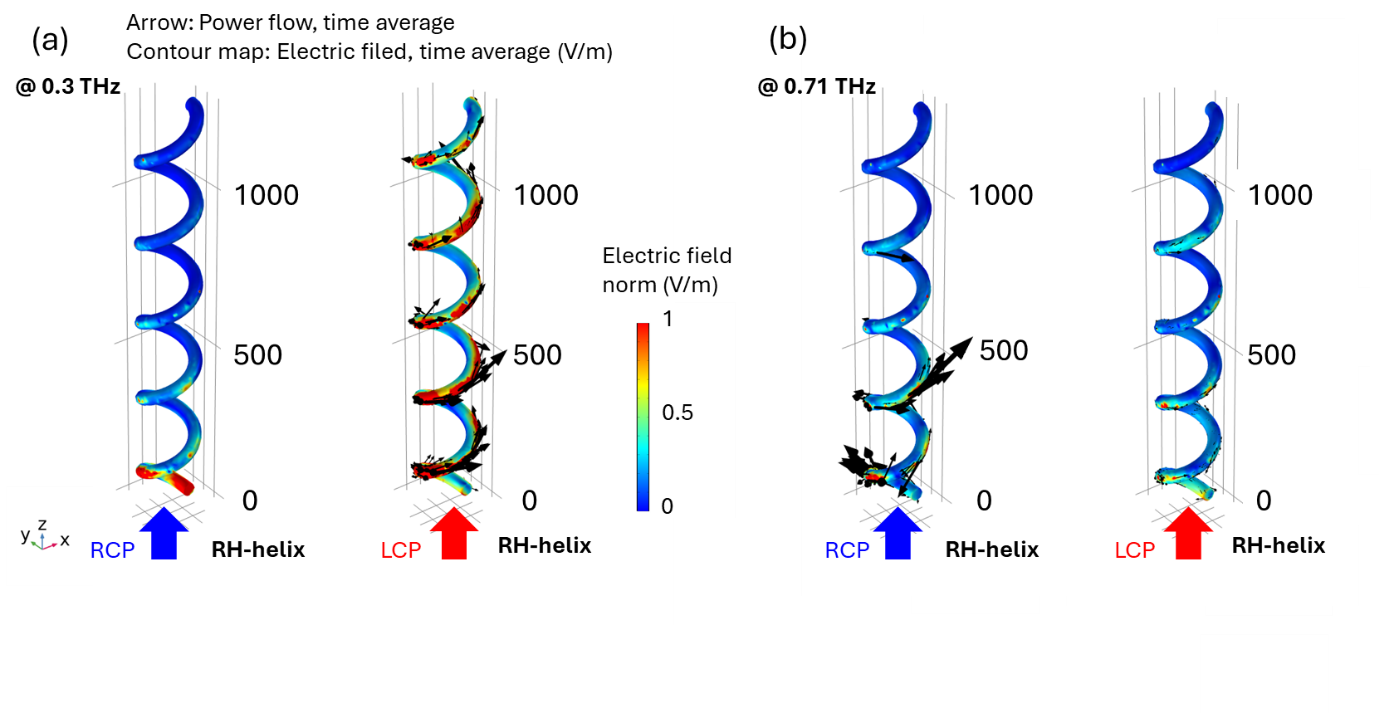


**Figure S4.** Near-field distribution map of the right-handed (RH) helix at (a) 0.3 THz and (b) 0.71 THz, where the ellipticity shows negative and positive, respectively.


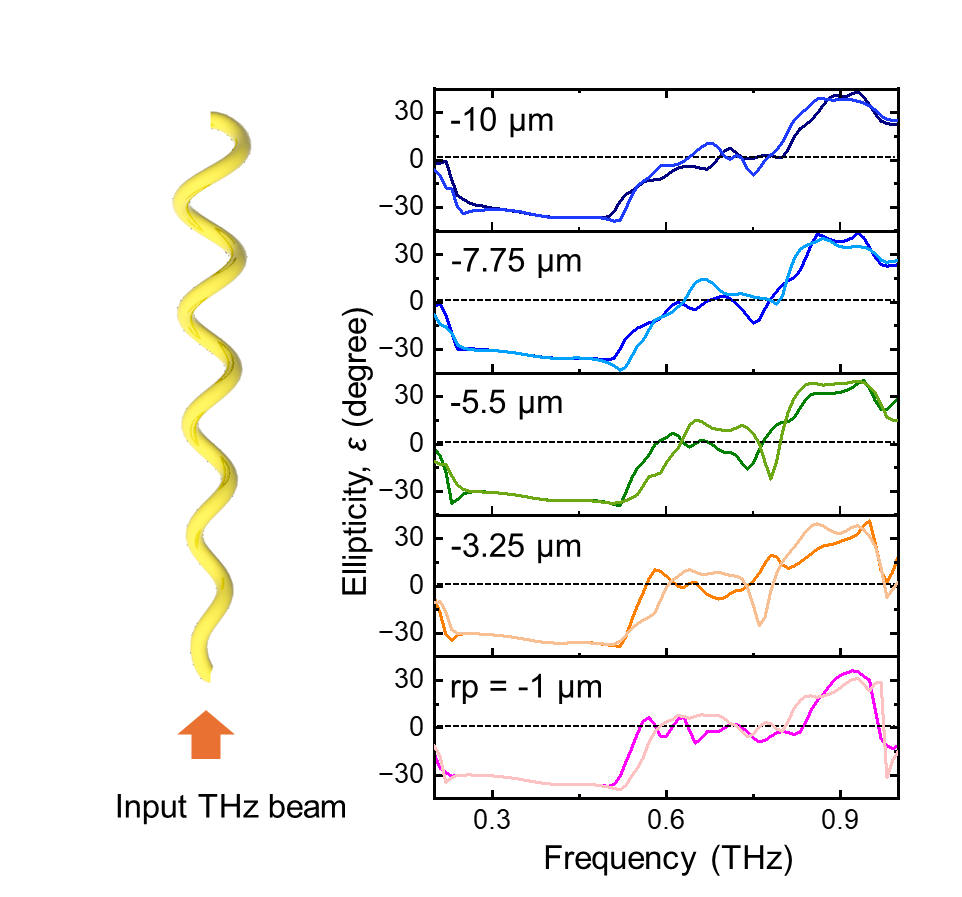


**Figure S5. Ellipticity results for helix with varying rp in an inverted configuration.** Results of helical arrays with *rp* = -1 μm to -10 μm are shown, using the same structure as in Fig.2l, but flipped with respect to the input beam. In this configuration, the beam is introduced with a smaller radius.


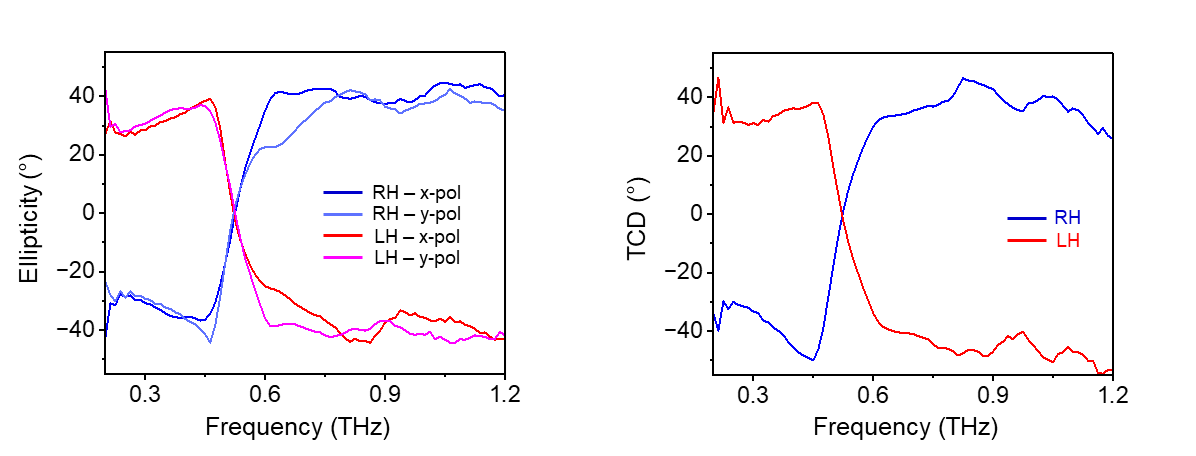


**Figure S6.** Measured ellipticity spectra for both right-handed and left-handed helical metamaterials, and calculated TCD.


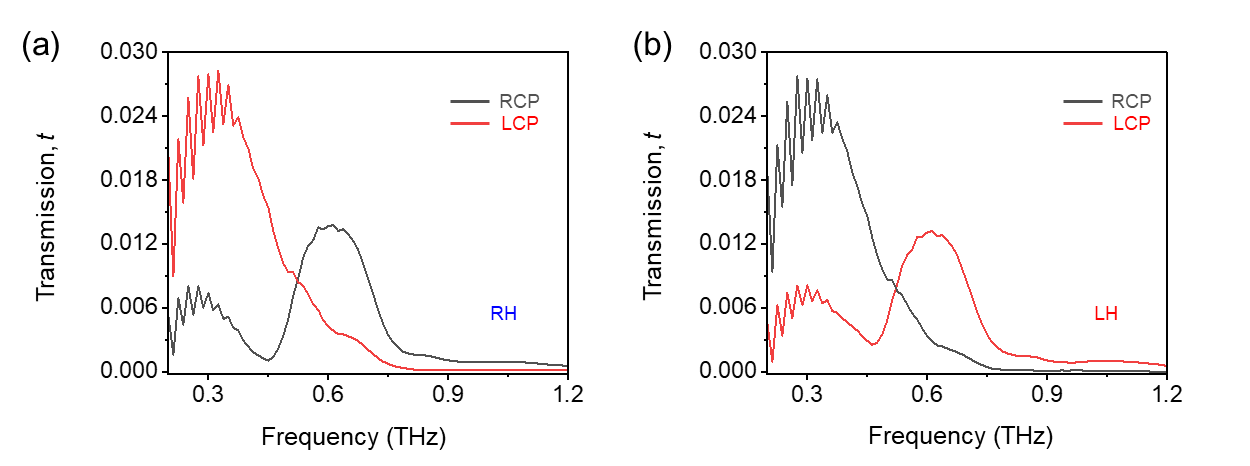


**Figure S7.** Measured absorption coefficient of PDMS, showing a low absorption (< 5 cm⁻¹) and a dispersionless characteristic across the entire frequency range.


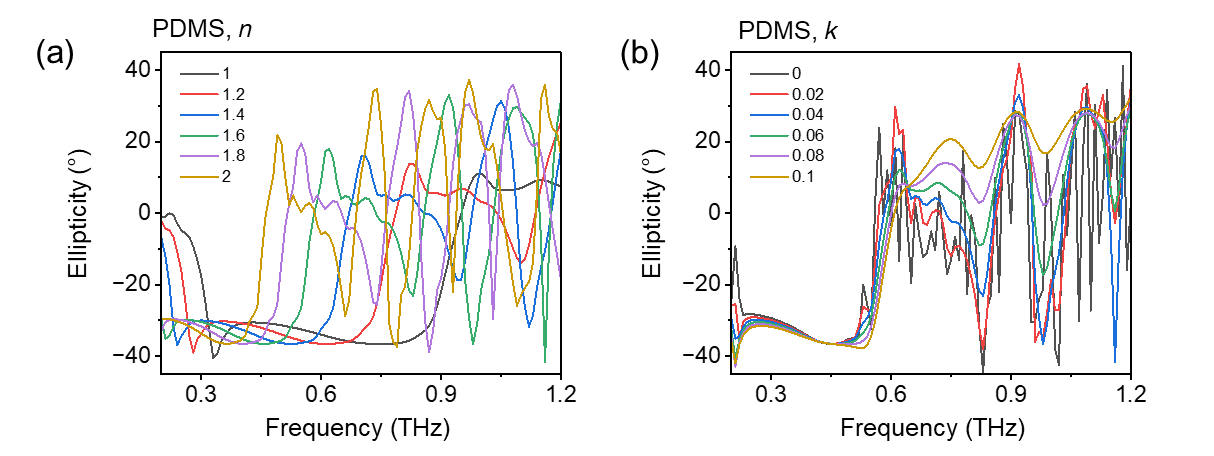


**Figure S8.** Simulated ellipticity of RH helix structure embedded in PDMS, with varying values of (a) refractive index, *n*, and (b) extinction coefficient, *k*.


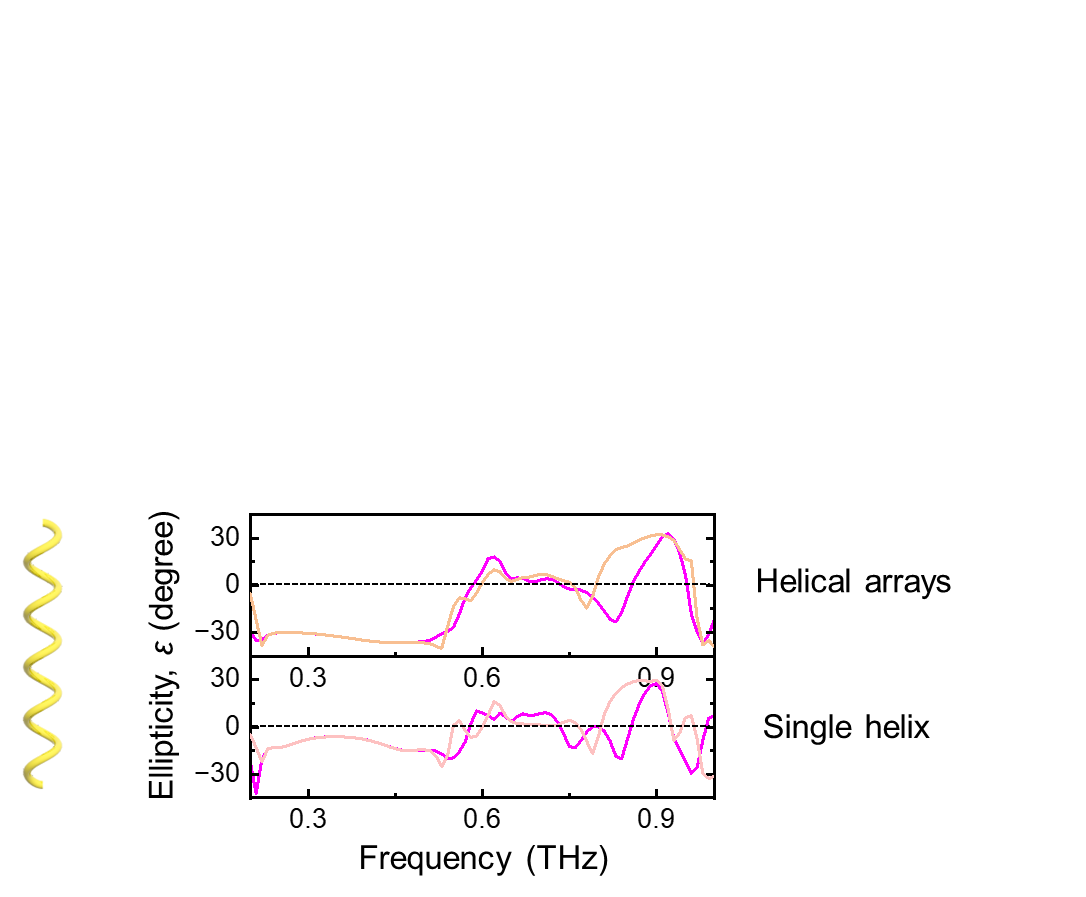


**Figure S9. Ellipticity comparison between a single helix and helical arrays with a 2D square lattice.** Helices with parameters of *ap* = 250 μm, *nt* = 5, *mr* = 75 μm, *wr* = 20 μm, and *rp* = 0 μm were chosen for the comparison. The result show that the overall shape and peak positions are quite similar, while the magnitudes are stronger when the helix is arrayed.

**Supplementary Movies S1 and S2.** The evolution of the induced electric field distribution is depicted on the surface of the helices using color, while the field in the surrounding space is represented by arrows, shown with respect to the frequency.

**REFERENCES**

[1] T. Ma, Q. Huang, H. He, Y. Zhao, Xi. Lin, Y. Lu, *Opt Express* **2019**, *27*, 16624.

[2] R. Alaee, C. Rockstuhl, I. Fernandez-Corbaton, *Opt Commun* **2018**, *407*, 17.

[3] L. Carletti, A. Locatelli, O. Stepanenko, G. Leo, C. De Angelis, *Opt Express* **2015**, *23*, 26544.

[4] W. J. Choi, G. Cheng, Z. Huang, S. Zhang, T. B. Norris, N. A. Kotov, *Nat Mater* **2019**, *18*, 820.

[5] W. J. Choi, S. H. Lee, M. Cha, N. A. Kotov, *Advanced Materials* **2024**, *36*, 2401131.
